# Analgesia for neuropathic pain by dorsal root ganglion transplantation of genetically engineered mesenchymal stem cells: initial results

**DOI:** 10.1186/s12990-015-0002-9

**Published:** 2015-02-12

**Authors:** Hongwei Yu, Gregory Fischer, Allison D Ebert, Hsiang-En Wu, Xiaowen Bai, Quinn H Hogan

**Affiliations:** Department of Anesthesiology, Medical College of Wisconsin, 8701 Watertown Plank Rd, Milwaukee, WI 53226 USA; Department of Cell Biology, Neurobiology & Anatomy, Medical College of Wisconsin, 8701 Watertown Plank Rd, Milwaukee, WI 53226 USA; Zablocki Veterans Affairs Medical Center, 5000 W National Ave, Milwaukee, WI 53295 USA

**Keywords:** Mesenchymal stem cells, Glial cell line-derived neurotrophic factor, Neuropathic pain, Dorsal root ganglion, Lentivector

## Abstract

**Background:**

Cell-based therapy may hold promise for treatment of chronic pain. Mesenchymal stem cells (MSCs) are readily available and robust, and their secretion of therapeutic peptides can be enhanced by genetically engineering. We explored the analgesic potential of transplanting bone marrow-derived MSCs that have been transduced with lentivectors. To optimize efficacy and safety, primary sensory neurons were targeted by MSC injection into the dorsal root ganglia (DRGs).

**Results:**

MSCs were transduced using lentivectors to express enhanced green fluorescent protein (EGFP) or to co-express the analgesic peptide glial cell line-derived neurotrophic factor (GDNF) and EGFP by a viral 2A bicistronic transgene cassette. Engineered MSCs were injected into the 4^th^ lumbar (L4) and L5 DRGs of adult allogeneic rats to evaluate survival in the DRGs. MSCs were detected by immunofluorescence staining up to 2–3 weeks after injection, distributed in the extracellular matrix space without disrupting satellite glial cell apposition to sensory neurons, suggesting well-tolerated integration of engrafted MSCs into DRG tissue. To examine their potential for inhibiting development of neuropathic pain, MSCs were injected into the L4 and L5 DRGs ipsilateral to a spinal nerve ligation injury. Animals injected with GDNF-engineered MSCs showed moderate but significant reduction in mechanical allodynia and hyperalgesia compared to controls implanted with MSCs expressing EGFP alone. We also observed diminished long-term survival of allografted MSCs at 3 weeks, and the development of a highly-proliferating population of MSCs in 12% of DRGs after transplantation.

**Conclusions:**

These data indicate that genetically modified MSCs secreting analgesic peptides could potentially be developed as a novel DRG-targeted cell therapy for treating neuropathic pain. However, further work is needed to address the challenges of MSC survival and excess proliferation, possibly with trials of autologous MSCs, evaluation of clonally selected populations of MSCs, and investigation of regulation of MSC proliferation.

## Introduction

Cell-based therapy has been proposed as a novel approach for treating painful peripheral neuropathy [1,2]. Transplantation of mesenchymal stem cells (MSCs) has been demonstrated to be a potentially therapeutic approach for the alleviation of chronic pain from various etiologies [2-8]. Prior studies have reported pain relief in rodent chronic pain models using systemic administration of MSCs [4,8], but this delivery approach requires large preparations of transplantable cells, depends on a nebulously defined propensity of MSCs to home in on injured tissue, and leads to MSCs trapping in the lung, liver, resulting in off-site tissue damage [9,10], making targeted delivery preferable. Injection of MSCs into the cerebral ventricle or subarachnoid space of mice and rats reduces neuropathic pain from nerve injury [11,12]. However, involving the central nervous system imposes difficulties for safe translation to human use. In contrast, dorsal root ganglia (DRGs), which harbor the sensory neuron somata, tolerate injections in rodents and humans without harm [13,14]. Although the DRG is an important site of posttraumatic pain pathology [15,16], it has not been developed as a site of cell therapy for pain. A DRG-targeted MSCs delivery approach has the benefits of tissue-specific delivery, reduced systemic side effects, and small total load of implanted cells.

A further enhancement of MSC-based therapy can be achieved by incorporating genetic engineering. Although native MSCs secrete a broad range of anti-inflammatory and neuromodulatory factors, only very low levels are produced [17,18]. Genetic manipulation provides delivery of high concentrations of therapeutic peptides selected for specific treatment of chronic pain resulting from different etiologies [19]. Since the MSCs are transduced *in vitro*, the benefits of genetic-based therapy are provided without exposing the patient to viral vectors. In trials using MSCs to treat inflammatory conditions in rat models, including painful arthritis, MSCs modified to enhance secretion of anti-inflammatory products resulted in successful treatment, whereas unmodified MSCs failed [20,21]. Since expression levels of therapeutic peptides by MSCs can be increased as much as 2,000-fold by viral transduction [20], genetic modification provides the opportunity to achieve effective treatment with a very much smaller dose of cells.

To test whether segmental therapy with engineered MSCs can relieve neuropathic pain, we examined the efficacy and limitations of DRG transplantation of MSCs following experimental nerve injury. To provide enhanced analgesic efficacy and *in vivo* tracking of transplanted cells, a lentivector was constructed containing a viral 2A ribosomal skipping domain to genetically modify MSCs for co-expressing two proteins [22]. Glial cell line-derived neurotrophic factor (GDNF) was chosen as the secreted analgesic factor since it has well established and potent analgesic properties [23-25], while enhanced green fluorescent protein (EGFP) was chosen for cell identification and tracking. *In vivo* viability of MSCs and their effectiveness in pain relief were evaluated by injection of these genetically engineered cells into the fourth and fifth lumbar (L4 and L5) DRGs of rats at the time of peripheral nerve injury induced by spinal nerve ligation (SNL).

## Methods

### Animals

Male Sprague Dawley rats (5–6 weeks old; 125–150 g body weight) were purchased from Charles River Laboratories (Wilmington, MA). All animal procedures were reviewed and approved by the Animal Care Committee of the Zablocki VA Medical Center Animal Studies Subcommittee and Medical College of Wisconsin IACUC (Permission number: 3690–03). Rats were housed in standard 12-hour cycle lighting and were allowed *ad libitum* access to food and water prior to and throughout the experimental protocol.

### Cell culture

Rat MSCs isolated from bone marrow of Sprague Dawley (SD) rats at ≤ 8 weeks after gestation, were obtained from Life Technologies (Carlsbad, CA, Lot No. 090716W01). According to the vendor, these were frozen at 4^th^ passage, and express flow-cytometry cell surface markers CD29, CD44, CD90, and CD106 (>70%) but are negative for CD11b, CD34, and CD45 (<5%). Their ability to differentiate into osteocytes, adipocytes, and chondrocytes has been experimentally validated [26,27]. We therefore used the cells for the subsequent experiments without further characterization. Cells were cultured in low-glucose α-MEM glutamax supplemented with 10% MSC-qualified FBS and 1X antibiotic-antimycotic mixture (Life Technologies) and were maintained in humidified incubators at 37°C with 5% CO_2_. Upon reaching 70 ~ 80% confluency, adherent cells were passaged by use of TrypLE Express (Life Technologies). MSCs were expanded from 6 to 10 passages for all experiments. Pheochromocytoma-derived (PC12) and HEK293T cell lines were obtained from the American Type Culture Collection (ATCC, Manassas, VA) and were cultured in standard conditions.

### Lentiviral constructs and infection

Lentiviral transfer plasmids pEF1α-EGFP and pEF1α-GDNF were used to express EGFP and GDNF, respectively, as prior described [28]. A viral 2A bicistronic lentiviral plasmid for co-expressing rat GDNF and EGFP under the EF1α promoter was constructed. Specifically, rat GDNF cDNA coding sequence (GenBank accession number, NM_199231) with omission of stop code was inserted into plasmid pEF1α-EGFP immediate downstream of EF1α promoter and a viral 2A autocleavage (or ribosome-skipping) sequence from *Thoseaasigna* virus 2A was then cloned in frame between GDNF and EGFP to generate pEF1α-GDNF-2A-EGFP. Lentivectors (LV) expressing EGFP (LV-EGFP) and GDNF (LV-GDNF) or co-expressing GDNF and EGFP (LV-GDNF-2A-EGFP) were packaged using pEF1α-EGFP, pEF1α-GDNF and pEF1α-GDNF-2A-EGFP with packaging plasmid pCMVΔR8.74 and envelop plasmid pVSV-g, followed by lentiviral particle concentration by ultracentrifugation, and viral titration by fluorescence-activated cell sorting (FACS) or qPCR, as previously reported [28]. The titers were in the range of 1 × 10^8^ to 1 × 10^9^ TU/ml. Cultured MSCs grown to 50% confluence were infected by LV-EGFP or LV-GDNF-2A-EGFP in the presence of 8 μg of polybrene (Sigma-Aldrich, St Louis, MO) per ml at multiplicities of infection (MOI) = 20. After infection at 37°C for 10 h, the medium was replaced. Mock transduction was performed under the same conditions but without added virus. Transduction efficiency was estimated under a fluorescent microscope by calculating the percentage of green cells out of total 200 counted cells. PC12 cells were transduced using LV-EGFP, LV-GDNF, or LV-GDNF-2A-EGFP, respectively, following the same procedure.

### Measurement of GDNF secretion in cell cultures

To measure GDNF secretion by PC12 cells and MSCs transduced by various LV constructs, equal numbers (1x10^5^) of non-transduced cells and LV transduced stable cells were plated at 50% confluence. The media were collected after 72 h and the concentration of GDNF in culture media were analyzed using an ELISA kit (Promega, Madison, WI) conducted in 96-well microplates according to the manufacturer’s instructions.

### Immunoblots

Cell lysates were analyzed to determine transgene expression as described previously [28]. DRGs were harvested 3 weeks after transplantation with MSCs transduced with LV-GDNF-2A-EGFP, and transgene expression (GDNF-2A and EGFP) was determined by immunoblots using the optimized GDNF antibody with a dilution that can only detect GDNF-2A derived from the transplanted cells but not endogenous GDNF. Briefly, 20μg of protein from DRG homogenates was loaded onto SDS–PAGE, transferred, and probed with a monoclonal anti-GFP antibody (1:1000, Santa Cruz Biotechnology, SCB, Santa Cruz, CA) or rabbit polyclonal anti-GDNF antibody (1:1000, SCB). Immunoreactive proteins were detected by enhanced chemiluminescence (Pierce, Rockford, IL, USA) after incubation with HRP-conjugated anti-mouse or anti-rabbit IgG (1:2000, SCB). α-Tubulin (TUBA) was used as a loading control.

### Cell transplantation into DRGs

Cultured engineered MSCs were detached by incubation with TrypLE and centrifuged at 400 *g* for 5 min, after which the pelleted MSCs were resuspended in the sterilized PBS, viability determined by trypan blue, and cell numbers counted by hemocytometer and adjusted to 10^7^ per ml. Since both SNL and DRG injection of MSCs involves surgery at the same site, the procedures were performed at the same time in order to avoid the added trauma and uncertain results of performing one or the other in the setting of local scaring and adherent tissues. DRG injection of MSCs into allogeneic rat recipients was performed as described previously with minor modification [13]. Briefly, the lateral aspects of the L4 through L6 vertebrae were surgically exposed, and a minimal foraminotomy was performed to expose the distal pole of the L4 and L5 DRGs. An injection volume of 2μl per ganglion was chosen on the basis of previous findings [13]. A total load of 2×10^4^ cells per DRG was injected based on a pilot experiment with injections of 1×10^4^ to 3×10^4^ cells, which showed that this number provided widespread distribution in DRG 1 week after injection. Injection was performed through a pulled glass micropipette with a tip diameter of 60-80 μm, which allowed resuspended MSCs to be injected through a micropipette without mechanical damage (data not shown). The surgical incision was closed in layers with absorbable suture and skin staples, which were removed after 7–10 days.

### Immunohistochemistry (IHC) and imaging

IHC staining on paraffin-embedded sections was performed by a standard fluorescent IHC protocol, as previously described [28]. Sections were immunolabeled with the primary antibodies of monoclonal GFP (1:400, SCB) or rabbit polyclonal GFP (1:400, Cell Signaling, Danvers, MA), monoclonal β3-tubulin (TUBB3, 1:500, SCB), rabbit anti-glutamine synthetase (GS, 1:600, SCB), monoclonal proliferating cell nuclear antigen (PCNA, 1:200, SCB), rabbit anti Bcl-associated X protein (Bax, 1:100, SCB), rabbit polyclonal GDNF (1:400, SCB), monoclonal STRO-1 (1:200, Life Technologies), rabbit polyclonal glial fibrillary acidic protein (GFAP, 1:4000, Dako, Carpinteria, California), and α-smooth muscle actin (SM actin, Sigma-Aldrich, 1:1000), with BSA replacement of the first antibody as the negative control. The appropriate fluorophore-conjugated (Alexa 488 or Alexa 594) secondary antibodies (Jackson ImmunoResearch, West Grove, PA) were used to reveal the primary antibodies. The sections were examined and images captured using a Nikon TE2000-S fluorescence microscope (El Segundo, CA) with filters suitable for selectively detecting the green and red fluorescence, and a QuantiFire digital camera (Optronics, Santa Barbara, CA).

### Experimental peripheral nerve injury and behavioral testing

Peripheral nerve injury by the SNL model [29] was induced in isoflurane-anesthetized animals with tight ligation of the right L5 spinal nerves between the DRG and the beginning of the spinal nerve. Sensory testing was performed in a blinded fashion. Mechanical allodynia was assessed by von Frey test using calibrated filaments with the up-down method [30]. Mechanical hyperalgesia was assessed by noxious punctate mechanical stimulation (pin test) [31], in which a 22g spinal anesthesia needle was applied to the hind paws with enough force to indent, but not puncture, the skin. Stimulations were applied 5 times to each hindpaw, in an alternating pattern. This was then repeated after at least 2 minutes, for a total of 10 stimulations. Response was either a brief, simple withdrawal of the paw with an immediate return to the cage floor, or a response characterized by sustained lifting and grooming of the affected paw, possibly with shaking, lasting at least 1 second. The latter behavior has been termed hyperalgesia behavior, and is associated with an aversive experience in the context of peripheral nerve injury [32]. The number of instances of hyperalgesia behavior at each timepoint (out of 10 stimulations) was recorded.

### Statistics

For comparison of behavior between groups, data were converted to area under the curve for each animal. Planned comparisons were tested between groups by *t*-test and were corrected for multiple comparisons with Bonferroni correction. Statistics were performed using GraphPad Prism 6.03 (GraphPad Software La Jolla, CA). Results are reported as mean and standard deviation. A probability of p < 0.05 was considered as significant.

## Results

### Efficient generation of stable GDNF-secreting and EGFP-labeled MSCs by a bicistronic lentivector

**Figure 1:**
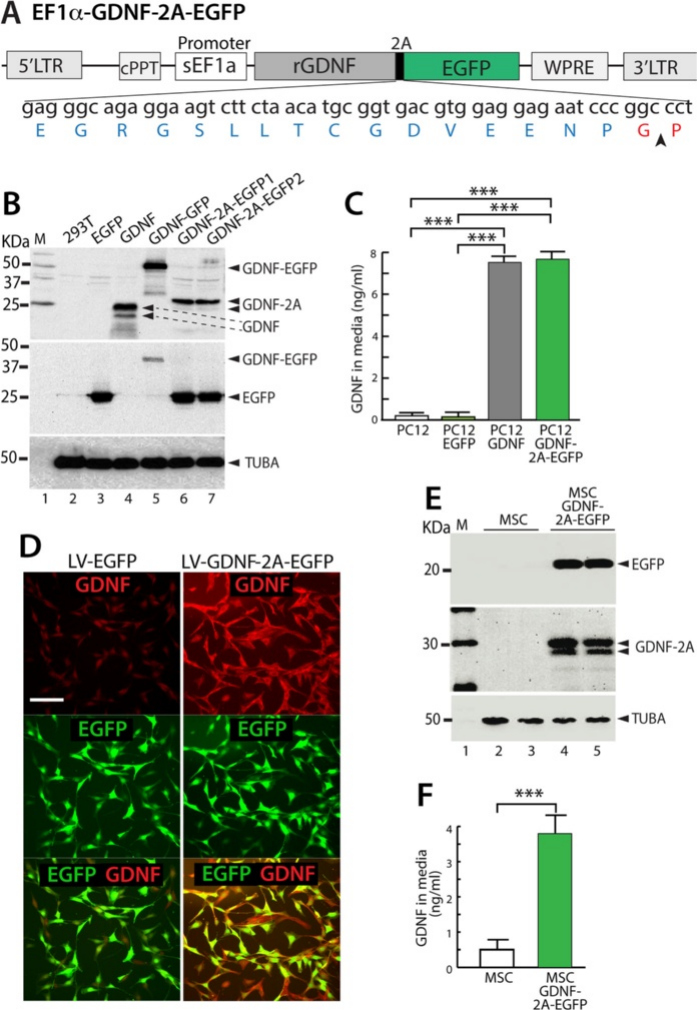
**Generation of GDNF-secreting MSCs. (A)** Schematic diagram illustrates the key elements of the lentiviral plasmid containing a viral 2A bicistronic transgene cassette (2A peptide depicted below with an arrowhead denoting the cleavage site) to co-express EGFP and GDNF driven by a short EF1α (sEF1α) promoter. *Cis*-acting sequences contain cPPT (central polypurine tract) and WPRE (woodchuck posttranscriptional regulatory element). **(B)** Immunoblots of HEK293T lysates for GDNF (top panel) or EGFP (middle panel) transfected with the lentiviral plasmids carrying the following transgenes: HEK293 (lane 2), EGFP (lane 3), GDNF (lane 4), GDNF-EGFP fusion (lane 5), and two GDNF-2A-EGFP constructs (lane 6,7). Various protein bands are denoted along the right side of the panels. Native GDNF appears as two bands due to the variable retention or cleavage of the signal peptide portion. The bands for GDNF-2A are approximately 5KDa heavier than GDNF due to retention of the 2A peptide up to the cleavage site. Alpha-tubulin (TUBA) was used as the protein loading control (bottom panel). **(C)** PC12 cells were transduced using lentivectors (LV) expressing EGFP, GDNF, or GDNF-2A-EGFP, respectively. ELISA analysis of media demonstrates an efficient GDNF secretion from the cells transduced with LV-GDNF-2A-EGFP, similar to LV-GDNF transduction, whereas secretion is minimal in controls (PC12 or PC12 transduced with LV-EGFP). n = 4 for each group, ***p < 0.001. **(D)** Immunocytofluorescence shows minimal GDNF expression in MSCs transduced by LV-EGFP, while MSCs transduced by LV-GDNF-2A-EGFP develop substantial GDNF expression 3 days after transduction (scale bar = 100 μm). **(E)** Immunoblots of cell lysates of MSCs transduced with LV-GDNF-2A-EGFP show EGFP and GDNF expression, which is absent in naïve MSCs. **(F)** ELISA of media shows that naïve MSCs secreted low levels of GDNF, which increased five-fold in the MSCs after LV-GDNF-2A-EGFP transduction, n = 4 for each group, ***p < 0.001.

In order to engineer MSCs to express two transgene products, we developed a lentivector LV-GDNF-2A-EGFP that contains a viral 2A bicistronic transgene cassette to co-express the analgesic peptide GDNF as well as EGFP (Figure 1A). Simultaneous expression of both genes and cleavage efficiency was confirmed in 293T cells by immunoblots of lysates transfected by LV-GDNF-2A-EGFP plasmids, with GDNF and GNDF-EGFP fusion plasmid transfections as controls (Figure 1B). Using this cassette, GDNF protein secretion was evaluated in neuron-like PC12 cells transduced with LV-GDNF-2A-EGFP, compared with transduction by LVs encoding only GDNF or EGFP. The media collected from these cultures demonstrated efficient GDNF secretion using ELISA analysis (Figure 1C). These results demonstrate the ability to deliver proteins with different final destinations from a single 2A bicistronic construct, i.e., secreted GDNF peptide for therapy and intracellular EGFP label for identifying transplantable cells. We next tested the ability of MSCs transduced by LV-GDNF-2A-EGFP to produce stable GDNF secretion and EGFP expression in MSCs. Both immunohistochemistry (Figure 1D) and immunoblot of the cell lysates (Figure 1E) demonstrated expression of both EGFP and GDNF by the LV-GDNF-2A-EGFP transduced MSCs, while no GDNF was found in cells or lysate of naïve MSCs. ELISA (Figure 1F) showed that LV-GDNF-2A-EGFP transduced cells secreted GDNF at a 5-fold greater rate than native MSCs.

### Survival and integration of engineered MSCs in DRG

We first determined if MSCs can survive after transplantation into DRGs of adult rats. Whereas fluorescent dyes such as DAPI are not a reliable marker for MSCs *in vivo* [33], GFP expression provides definitive tracking of surviving MSCs [34]. Therefore, we transduced the MSCs with LV-EGFP (hereafter referred to as EGFP-MSCs) or with LV-GDNF-2A-EGFP (hereafter referred to as GDNF/EGFP-MSCs). The transduction of MSCs by lentivectors was optimized at MOI = 20, at which level more than 90% of MSCs were successfully transduced. To assess MSC survival, differentiation, and integration into DRGs, we transplanted engineered allogeneic cells into the L4 and L5 DRGs in adult non-injured rats. The recipient rats were euthanized at 1, 2, 3 and 4 weeks post-transplantation and DRG sections were analyzed by immunofluorescence staining of transgenes.

### EGFP-MSCs

**Figure 2:**
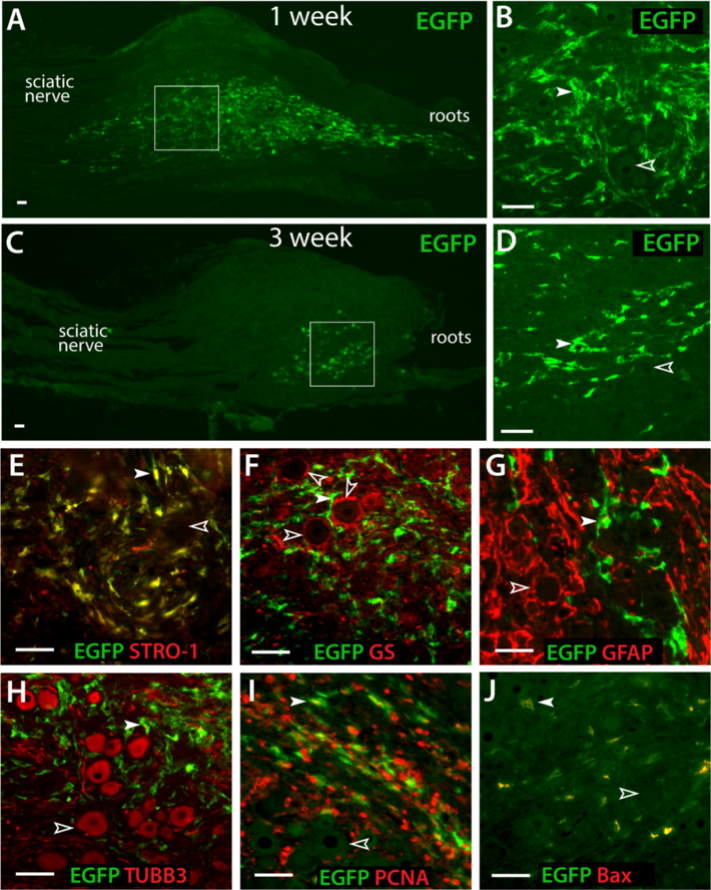
**Survival and differentiation of transplanted EGFP-MSCs in the non-injured DRGs.** Immunohistochemical preparations reveal transplanted EGFP-MSCs in DRG sections, showing numerous EGFP-MSCs with typical fibroblast-like morphology and some MSC migrating in a short distance along roots and sciatic nerve 1 week after transplantation (**A**, outlined area magnified in **B**), but reduced numbers of engrafted EGFP-MSCs 3 weeks after transplantation **(C, D)**. Here and in subsequent panels, filled arrowheads point to the engrafted EGFP-MSCs, and empty arrowheads indicate EGFP-negative neurons or satellite glia. Transplanted MSCs retained STRO-1 expression typical of MSCs **(E)**. EGFP-MSCs (green) show a general distribution pattern in the extracellular matrix space, without disrupting the normal relationship in which satellite glia, stained here by GS (red), form rings enwrapping sensory neurons (unstained here, **F**). Identification of satellite glial cells by GFAP provides similar findings **(G)**. Transplanted EGFP-MSCs do not express GS **(F)** or GFAP **(G)**. Sensory neuron somata, labeled by TUBB3 (red), are typically separated from MSCs (stained with EGFP) that are negative for TUBB3 **(H)**. Engrafted EGFP MSCs express immunopositivity for proliferating cell nuclear antigen (PCNA) **(I)** and pro-apoptosis regulator Bax **(J)**. Scale bar = 50 μm for all images. Sections were harvested 1 week after transplantation in panels **E**, **F**, **H**, and **J**, and were harvested 3 weeks after transplantation in panels **G** and **I**.

In DRGs harvested 1 week after injection of EGFP-MSCs, numerous transplanted EGFP-expressing MSCs were observed in DRG sections, showing typical fibroblast-like morphology (Figure 2A, B). In general, the transplanted EGFP-MSCs were distributed in the extracellular matrix space without disrupting the normal pattern of satellite glial cell apposition to sensory neurons, suggesting well-tolerated integration of engrafted MSCs into DRG tissue. No EGFP-MSCs were found by IHC in the contralateral DRGs or the spinal cord (data not shown). Although it is impossible to accurately count surviving cells because of their aggregation, it was clear that fewer EGFP-expressing MSCs were evident 3 weeks post-transplantation (Figure 2C, D), while they retained morphology similar to that at 1 week post-transplantation. No engrafted MSCs were found by IHC in the recipient DRGs 4 weeks after transplantation (n = 10 DRGs).

Immunofluorescence was performed to determine the differentiation potentials of transplanted MSCs within the environment of the DRG. EGFP-MSCs were immunopositive for STRO-1 (an MSC marker, Figure 2E). MSCs did not express satellite glial cell marker GS (Figure 2F) or GFAP (Figure 2G). No MSCs were found to be positive for β3-tubulin, a sensory neuron marker (Figure 2H). Consistent with *in vitro* cultured cells (data not shown)*,* engrafted MSCs also expressed proliferating cell nuclear antigen (PCNA, Figure 2I), and immunopositivity for pro-apoptosis regulator Bax [35] (Figure 2J), which together indicate active cell cycling. Overall, these results indicate that MSCs can survive within DRGs for 2–3 weeks after transplantation under the conditions of this experiment, and that the surviving transplanted MSCs retained their primary MSC properties without spontaneous differentiation to neuronal or glial cell types in the DRG microenvironment during the period of observation.

### GDNF/EGFP-MSCs

**Figure 3:**
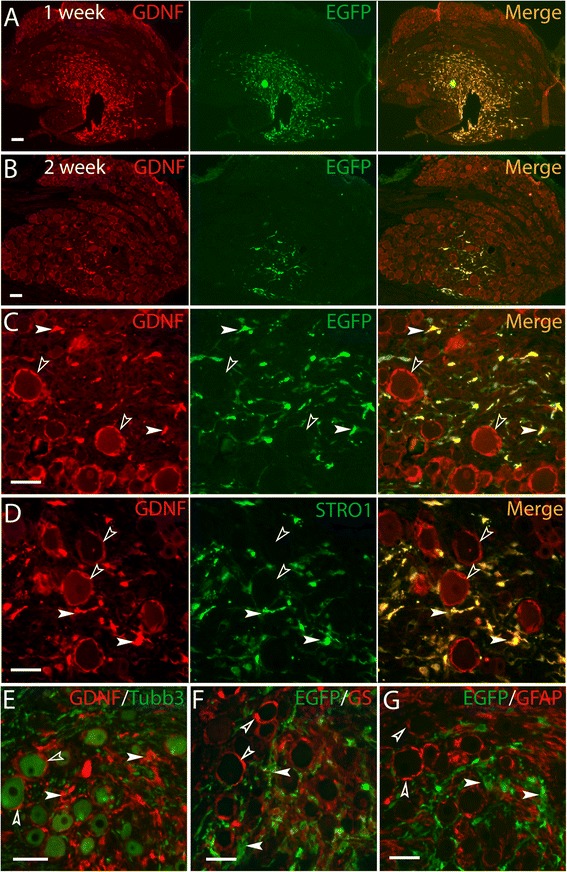
**Survival of transplanted GDNF/EGFP-MSCs in the non-injured DRGs.** Integrated GDNF/EGFP-MSCs are shown residing in extracellular spaces within DRGs at 1 week **(A)** and 2 weeks **(B)** post-transplantation using GDNF and GFP antibodies. High-power image **(C)** of DRG 2 weeks after transplantation reveals high expression of GDNF and GFP in fibroblast-like GDNF/EGFP-MSCs while the endogenous GDNF immunofluorescent (IF) signals are detected as ‘glia-rings’ around neurons and variably in neurons. Double immunostaining 2 weeks after transplantation shows that the grafted GDNF/EGFP-MSCs are immunopositive for STRO-1 **(D)** but immunonegative for Tubb3 **(E)**, GS **(F)**, and GFAP **(G)**. In C-G, the filled arrowheads denote engrafted GDNF/EGFP-MSCs, while the empty arrowheads indicate endogenous markers. Scale bar = 50 μm for all images.

We next evaluated survival of GDNF-secreting MSCs (i.e. GDNF/EGFP-MSCs) after DRG transplantation. Although others have identified enhanced survival of MSCs in the presence of GDNF [36], our findings showed that GDNF-expressing MSCs had the similar survival pattern (Figure 3A, B) as was seen for MSCs expressing EGFP alone. Immunostaining (Figure 3A-G) similarly revealed that GDNF expression did not affect MSC morphology, distribution profile and marker expression after transplantation.

### Analgesia from MSC engraftment

**Figure 4:**
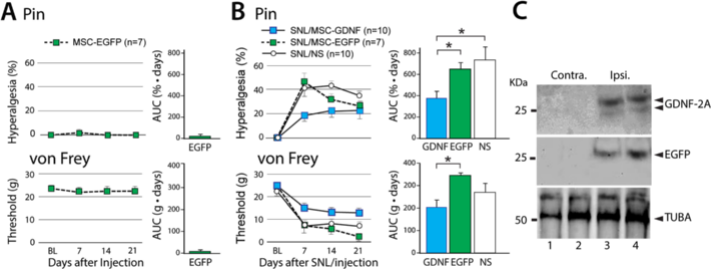
**Analgesic effects from GDNF-secreting MSC engraftment. (A)** In the absence of injury, transplantation of EGFP-MSCs did not change the frequency of hyperalgesia response upon fully noxious stimulation by Pin (top panel) or the threshold for withdrawal from mechanical stimulation with von Frey fibers (bottom panel). **(B)** In the setting of neuropathic pain from L5 SNL, animals transplanted with either type of cells (EGFP-MSCs or GDNF/EGFP-MSCs) at the time of nerve injury all developed mechanical hyperalgesia (Pin) and allodynia (von Frey) by 7 days later (left panels), which persisted for 21 days. However, those animals receiving GDNF/EGFP-MSCs showed a smaller reduction in withdrawal threshold, demonstrated by area under the curve (AUC) analysis comparing groups (right panel), *p < 0.05. **(C)** Immunoblots show detection of GDNF-2A (top panel) and EGFP (middle panel) derived from transplanted GDNF/EGFP-MSCs in DRGs (from 2 different animals) harvested 3 weeks after transplantation, while endogenous GDNF was not detected in control DRGs contralateral to the injury.

In the absence of injury, injection of EGFP MSCs caused negligible behavioral changes in either threshold for withdrawal from mechanical stimulation with von Frey fibers or the rate of hyperalgesia*-*type responses upon fully noxious stimulation by Pin (Figure 4A), suggesting preservation of normal mechanosensory neuron function in the presence of MSCs and minimal injury or inflammation due to the procedure. To test the efficacy of engineered MSCs as a treatment for neuropathic pain, animals were transplanted with either EGFP-MSCs or GDNF/EGFP-MSCs at the time of their SNL injury. Both L4 and L5 DRGs were injected since the etiology of pain after SNL may involve contributions from both the axotomized neurons of the L5 DRG as well as the intact adjacent L4 neurons [37]. Mechanical allodynia (von Frey) and hyperalgesia (Pin) developed by one week after nerve injury in both groups, which persisted for the 4-week duration of the experiment. However, those animals receiving GDNF/EGFP-MSCs developed a significantly smaller reduction in withdrawal threshold and smaller elevation in hyperalgesia responses compared to animals receiving EGFP-MSCs (Figure 4B). We additionally compared behavior of animals receiving SNL plus MSC transplantation to those receiving SNL with only saline injection into the DRGs, which showed that hyperalgesia was reduced with GDNF/EGFP-MSCs but not by EGFP-MSCs. To test for possible extended analgesia from GDNF/EGFP-MSC transplantation, we examined additional animals (EGPF-MSCs n = 5, GDNF/EGFP-MSCs n = 3) at 4 weeks, which showed no difference in either hyperalgesia (Pin, p = 0.11) or allodynia (von Frey, p = 0.36). MSCs were not evident by IHC in the serial sections from any DRGs in these animals. Together, these results indicate that MSCs expressing EGFP alone did not provide analgesia, whereas transplantation of MSCs engineered to secrete GDNF reduces pain behavior after nerve injury. Immunoblots using GDNF and GFP antibodies, collected at 3 weeks after transplantation, validated the production of GDNF by transplanted cells in the DRGs injected with GDNF/EGFP-MSCs, whereas the contralateral control DRGs showed no endogenous GDNF using optimized immunoblotting conditions (1:1000 dilution for GDNF antibody, Figure 4C).

### Overgrowth of transplanted MSCs in DRG

**Figure 5:**
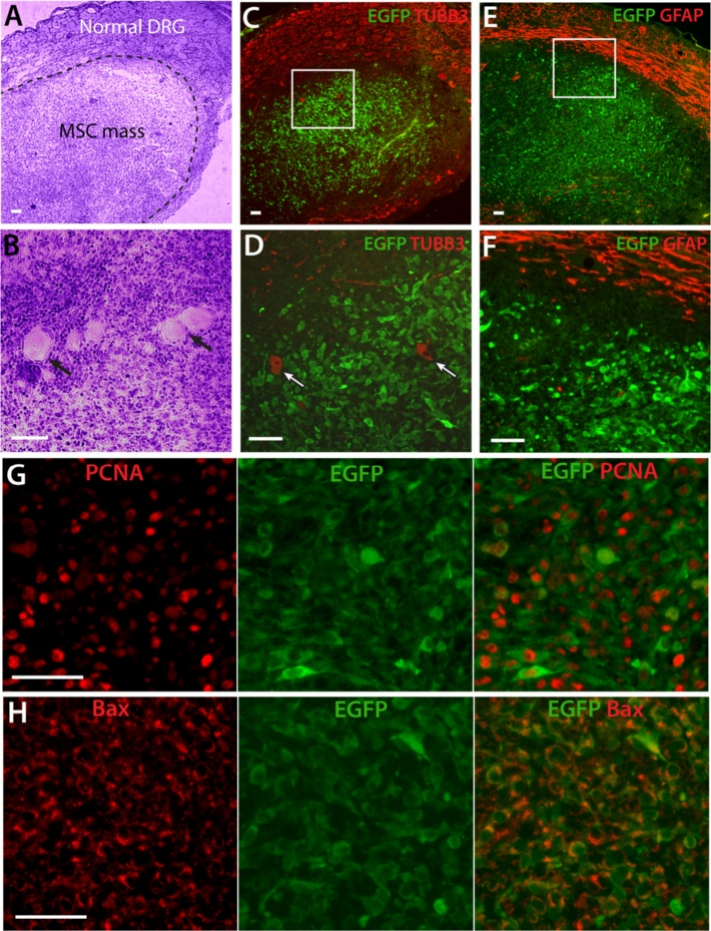
**Tumor-like aggregation of transplanted MSCs within DRG. (A, B)** Representative images from one of the DRGs in which MSC transplantation led to development of a mass show hematoxylin & eosin (H&E) stains of a tumor-like mass in a DRG from a EGFP MSC recipient rat (SNL), revealing a hypercellular aggregations of MSCs consisting of pleomorphic cells, with normal sensory neuron somata and axons mostly surrounding the mass **(A)** but also some dispersed within the MSC stroma (**B**, arrows). Immunohistochemical examination shows the cellular nodule is composed of EGFP-positive cells that are negative for TUBB3 (**C**, outlined area magnified in **D**, arrows point TUBB3-positive neurons) and GFAP (**E**, outlined area magnified in **F**). These MSCs express high level of PCNA **(G)** and immunopositivity for Bax **(H)**. Scale bars: **A-F** = 100 μm, **G, H** = 50 μm.

Accumulation of engrafted MSCs was observed in 12% of DRGs subjected to MSC transplantation and SNL (n = 8 out of 66 DRGs), forming a nodule within DRGs 3 or 4 weeks after injection with either EGFP-MSCs (n = 1 axotomized L5 DRGs and n = 2 adjacent L4 DRGs) or GDNF/EGFP-MSCs (n = 3 L5 and n = 2 L4 DRGs). Sensory evaluation of these 8 rats, which were excluded from the behavior analysis, showed anesthesia during mechanical stimulation in four of them, suggesting sensory function was adversely affected by the MSC mass. Hematoxylin & eosin staining of DRG sections (Figure 5A, B) revealed a hypercellular accumulation of MSCs within the DRG, consisting of pleomorphic cells with normal sensory neurons and axons surrounding the cellular mass or dispersed in the MSC stroma. Immunohistochemical examination revealed that the cellular nodule is composed of EGFP-positive cells that exhibit generally typical morphology of MSCs and immunopositivity for STRO-1 but negative for TUBB3 (Figure 5C, D), GFAP (Figure 5E, F), and α-smooth muscle actin (not shown). These MSCs expressed PCNA (Figure 5G) and were immunopositive for Bax (Figure 5H), similar to the population of MSCs unassociated with mass formation (Figure 5I, J). These results indicate that the MSCs that compose the nodular mass retain the phenotype of primary MSCs without definite cell transformation, and are not immunohistochemically distinct from MSCs that did not excessively proliferate.

## Discussion

A multitude of mechanistic observations have emerged from research on chronic pain, but these discoveries have not led to successful therapies. Genetic treatment strategies may provide a critical breakthrough to take advantage of molecular discoveries on the underlying causes of chronic pain. While using viral vectors to deliver transgenes to neural tissue may lead to highly specific treatments, this approach has inherent limitations, including unavoidable risks of activating the host immune system, oncogenesis through insertional mutagenesis, virus reactivation, and of generation of replication-competent virus following administration. An alternate genetic approach without these limitations would be very valuable. We have evaluated the therapeutic potential of transplanting allogeneic MSCs into the DRG after they have been engineered to secrete an analgesic peptide, thereby functioning as biological mini-pumps for the treatment of neuropathic pain. Our findings show encouraging results and provide evidence that cell therapy at the level of the DRG, using genetically modified MSCs, may potentially be developed for treating chronic neuropathic pain.

A prior attempt at DRG transplantation of unmodified MSCs used a nuclear stain for identifying MSCs [38], but this technique allows transfer of the staining dye to host cells after MSC death [39], and sections revealed staining in a pattern probably representing dye uptake by satellite glial cells, while analgesia was seen only at 3 days after transplantation. In contrast, our labeling method by EGFP expression assures certain identification of the surviving engrafted MSCs, and additionally confirms transgene expression by the engineered cells. Thus, this report is the first to conclusively demonstrate the analgesic potential of cell-mediated analgesia by targeted DRG injection. We used a model of peripheral nerve injury that has relevance to clinical conditions such as surgical nerve injury at the time of amputation, in which early preemptive treatment may limit the development of neuropathic pain. In this study, we did not observe pain attenuation in injured rats with EGFP-MSC transplantation, while GDNF-secreting MSCs provided moderate pain suppression. This supports an interpretation that analgesia after DRG injection of GDNF-secreting MSCs is attributable to their production and secretion of GDNF.

We observed MSC survival for up to 3 weeks, although there is a fall-off of cell numbers. Since this treatment strategy for chronic pain requires MSC survival to provide continued secretion of the therapeutic peptide, cell loss could result in a decrement of analgesia. Therefore, survival of allografted MSCs would be an important obstacle for successful long-term cell-based therapy for chronic pain. Allogeneic MSCs have long been reported to be nonimmunogenic [40]. However, recent studies describe limited allogeneic MSC survival and generation of antibodies against allogeneic donor MSCs after transplantation into immunocompetent recipients [40-46]. This suggests that MSCs may not intrinsically be immuneprivileged, and that our observation of cell loss is attributable to immunological rejection. Other DRG environmental factors that may limit longevity of transplanted MSCs include peripheral nerve injury-induced inflammatory response, oxidative stress conditions, and presence of pro-apoptotic factors and chemokines, and factors during initial cell culture condition and passage number may also play a role [39,47]. These mechanisms may not be mutually exclusive, and together may influence the survival of transplanted cells. It is reported that clonally grown MSC subpopulations may be identified with greater survival [48]. Further studies could identify desirable biological properties and their markers in cultured MSC subpopulations before expansion and transplantation [49]. A promising alternative is the potential of substantially improving survival through the use of autotransplantation [50,51]. Since MSCs can be cultured from bone marrow or adipose tissue [52] and propagate rapidly, generating the relatively small population needed for transplantation into DRGs would be feasible in the clinical setting even when using MSCs originating from the patient’s own tissue.

Our data also show that engineering MSCs by LV transduction enabled them to secrete GDNF. Transplantation of these GDNF-secreting MSCs provided an antihyperalgesic effect compared to similarly injured animals receiving saline injection, while MSCs expressing EGFP alone did not, which indicates that the treatment effect is due to the secretion of GDNF, not simply the presence of MSCs *per se*. The therapeutic mechanism of GDNF may involve prevention of injury-induced ectopic neuronal activity by preventing shifts in the expression of voltage-gated Na^+^ channel subtypes in DRG neurons [23,53]. Prior findings using lentiviral-mediated GDNF expression [54] indicate that the predominant source of analgesia may be from actions of GDNF on the intact L4 neurons after SNL rather than the axotomized neurons of L5. This key role of the transgene also suggests that development of this treatment approach could employ a wide range of analgesic peptides such as inhibitory neurotransmitters (e.g. beta-endorphin [55], anti-inflammatory peptides (e.g. IL-10 [21], fractalkine [56]) neurotrophins (NT-3 [55], VEGF [57]), and soluble receptors (e.g. soluble tumor necrosis factor receptor [58]) to treat various chronic pain conditions.

It is unclear why others [38] have observed analgesia resulting from transplantation of non-engineered MSCs, in contrast to our findings. This may be attributable to the exact nature of the MSCs. For instance, culture conditions and passage number may be factors that affect the biological function of different cell preparations [49]. In support of this concept, intrathecal application neural stem cells has been shown to alleviate neuropathic pain in rats through release of GDNF [12], but another report shows that SNL-induced pain behavior in rats is not reduced by intrathecal 15-passage MSCs [59].

Biosafety is a critical concern that could limit development of MSC-based therapy for pain. Therefore, a problematic observation from this study is the development of an MSC mass in 12% of recipient DRGs at 3-4 weeks post-transplantation. While it is reported that MSCs secreting transgenic GDNF may have higher rates of engraftment and survival [36], we doubt that this was a factor in the production of a mass since we also observed accretions of MSCs that expressed only EGFP. The results showing both occasional formation of a highly proliferating cellular mass in some DRGs but also a generally limited MSC survival duration in the majority of recipient DRGs may reflect the heterogeneous nature of the MSCs used in this study. It is well-recognized that MSCs constitute a non-uniform population of stromal cells [60]. Additionally, *ex vivo* culture of MSCs can induce spontaneous genome instability and alterations of functional and biological properties in some lineages of MSCs, leading to buildup of genetic aberrations to become tumorigenic clones with a growth advantage [61]. Indeed, tumorigenesis of transplanted allogeneic MSCs in various targeted tissues has been observed [61-64]. Some reports of tumorigenesis have subsequently been attributed to cross-contamination of MSC cultures with tumor cell lines [65,66]. This factor did not likely contribute to the formation of MSC masses in our series since there were no exogenous tumor cells in the environment where the MSCs were cultured. Finally, there is a potential risk that lentivector genetic modification may induce transformation by activating oncogenes due to promoter insertion [67]. However, extensive studies have shown that lentiviral vectors have a low tendency to integrate in places that potentially cause tumor and are well suited for safe and effective clinical gene transfer [68,69]. Overall, MSC tumorigenicity provoked by lentivector-transduction has been considered to be low [48,70]. Since MSCs similar to those used in the present study did not form masses when implanted into the brain [39,71], the specific molecular microenvironment of the DRG, particularly following peripheral nerve injury, may be a factor contributing to occasional excessive propagation.

In conclusion, our initial findings demonstrate that segmental pain therapy with DRG transplantation of genetically modified MSCs can provide therapeutic benefit for neuropathic pain. Important challenges for developing this method include characterizing parameters that influence MSC cell cycle and proliferation, prolonging *in vivo* MSC survival, and preventing the occasional development of tumor-like aggregations of transplanted cells. With these advances, engineered MSCs may offer a novel opportunity for cell-based gene therapy that could provide safe, sustained segmental therapy for treating chronic pain.
